# NIPT detects occult maternal malignancies via cfDNA aberration profiling: a case report

**DOI:** 10.3389/fmed.2026.1759660

**Published:** 2026-04-29

**Authors:** Liqiong Wu, Xiaohua Wang, Jin An, Yaxian Liu, Ruifang Bai, Jie Wang, Wenbin Zhang

**Affiliations:** Department of Genetics, Inner Mongolia Maternity and Child Health Care Hospital, Hohhot, China

**Keywords:** cell-free DNA (cfDNA), copy number variation (CNV), hepatocellular carcinoma, maternal malignancy, Non-Invasive Prenatal Testing (NIPT)

## Abstract

Non-Invasive Prenatal Testing (NIPT), primarily used for fetal aneuploidy screening, can incidentally detect occult maternal malignancies through abnormal genomic patterns such as multiple chromosomal aneuploidies (MCAs) or copy number variations (CNVs). These findings, often discordant with fetal results, have led to diagnoses of various cancers, including breast cancer, lymphoma, and ovarian cancer. Although promising for early detection, this application is currently limited by low specificity for early-stage tumors and the absence of standardized clinical protocols. Multidisciplinary collaboration and the development of integrated predictive models combining cell-free DNA (cfDNA) analysis with serum biomarkers are essential to improve diagnostic accuracy and guide appropriate maternal follow-up.

## Introduction

Non-Invasive Prenatal Testing (NIPT), widely used for detecting fetal chromosomal aneuploidies such as Trisomy 21, analyzes cell-free fetal DNA in maternal blood. Recent studies indicate that unusual genomic patterns, especially multiple chromosomal aneuploidies (MCAs), may also suggest underlying maternal cancers. While this dual potential offers promising avenues for early cancer risk assessment during pregnancy, current evidence remains preliminary. Standardized protocols and multidisciplinary collaboration are needed to validate its clinical utility and address associated ethical challenges.

## Case presentation

**FIGURE 1:**
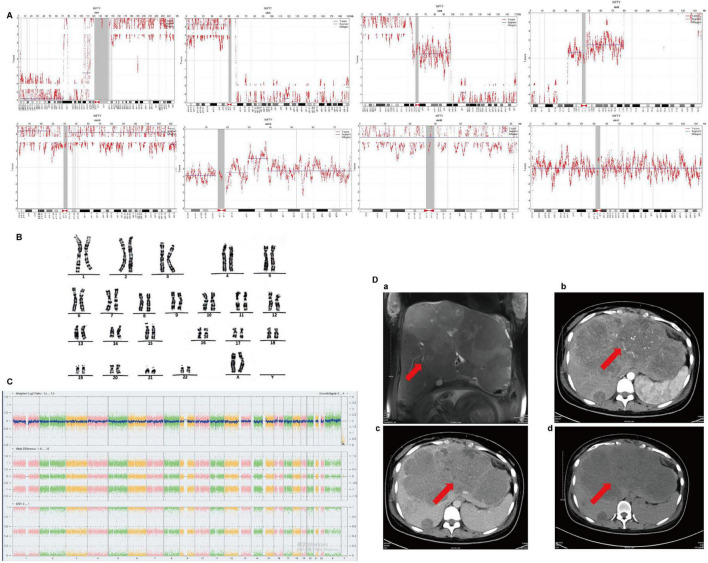
Incidental detection of maternal hepatocellular carcinoma through abnormal Non-Invasive Prenatal Testing (NIPT) findings. **(A)** NIPT reveals a complex maternal copy number variation (CNV) profile. Genome-wide CNV plot showing copy number gains and losses across chromosomes. Duplications are represented by signals above the baseline, indicating copy number gains in regions 1q21.1-q44 (∼102.9 Mb), 4p16.3-q11 (∼49.8 Mb), 6p25.3-p11.2 (∼60 Mb), 6q12-q16.2 (∼23.1 Mb), 8p12-p11.1 (∼12.5 Mb), 8q11.21-q21.12 (∼12.5 Mb), 10p15.3-p11.21 (∼33.7 Mb), 10q11.21-q26.3 (∼89.4 Mb), 18q12.1-q12.3 (∼11.3 Mb), 20p13-p11.21 (∼29.8 Mb), and 20q11.21-q13.33 (∼38.5 Mb). Deletions are represented by signals below the baseline, indicating copy number losses in regions 1p36.33-p31.3 (∼70 Mb), 4q13.1-q35.2 (∼87.6 Mb), 6q21-q27 (∼68.4 Mb), and 8p23.3-p21.1 (∼29 Mb). **(B,C)** Fetal testing confirms normal results. **(B)** Fetal karyotype analysis shows a normal female complement (46,XX). **(C)** Fetal chromosomal microarray analysis demonstrates no pathogenic copy number variants. **(D)** Magnetic resonance imaging (MRI) findings of multifocal hepatocellular carcinoma (HCC). MRI performed at 34 weeks of gestation (without gadolinium contrast due to pregnancy) reveals multiple solid liver lesions. (a) Coronal T2-weighted imaging shows hyperintense lesions. (b) Dynamic contrast-enhanced arterial phase and (c) portal phase imaging demonstrate characteristic enhancement patterns consistent with HCC. (d) Non-contrast T1-weighted imaging shows hypointense lesions.

Ethical approval number: [2025] Lunhan examination No. [014]. Ms. Wang, a 28-year-old woman (G2P1) with a history of hepatitis since childhood, underwent NIPT at 22 weeks of pregnancy. Two consecutive NIPT tests revealed severe genome-wide copy number variations and multiple chromosomal copy number variations in the sample (Figure 1A). Subsequent amniocentesis showed normal fetal karyotype (46, XX) and chromosomal microarray results (Figures 1B,C).

The patient was admitted at 34 weeks and 3 days of gestation due to upper abdominal pain and loss of appetite. On admission, physical examination showed a temperature of 37.0 °C, pulse rate of 97 beats per minute, respiratory rate of 20 breaths per minute, and blood pressure of 110/75 mmHg. She had palmar erythema but no obvious spider angiomas, localized distension in the right upper abdomen, and an irregular, firm, tender liver palpable three fingerbreadths below the costal margin with abdominal distension. Blood tests indicated abnormal liver function and elevated alpha-fetoprotein (AFP) levels (Table 1). Abdominal ultrasound revealed a singleton viable fetus in cephalic presentation during late pregnancy, along with hepatomegaly with multiple solid lesions. MRI confirmed hepatomegaly and multiple solid space-occupying lesions, suggestive of multifocal hepatocellular carcinoma (Figure 1D). Due to the unavailability of tumor tissue samples, this study did not experimentally validate copy number variations in the hepatocellular carcinoma tissue. Instead, NIPT DNA libraries were subjected to 30 × depth resequencing to further characterize the genomic aberrations. Sequencing was performed on the BGIseq-2000 platform (BGI, Shenzhen, China) with single-end 50 bp reads. Raw sequencing data were aligned to the human reference genome (hg38) using BWA-MEM. Copy number variation (CNV) analysis was performed using WisecondorX with a bin size of 100 kb. Genomic instability was defined as the presence of at least three large-scale (>5 Mb) CNVs involving at least two different chromosomes. Quality control parameters included a median absolute deviation of read depth < 0.25, a mapping rate > 98%, and a duplication rate < 10%. Both the initial and resequencing analyses consistently revealed a highly unstable genome-wide copy number variation (CNV) profile in the maternal cfDNA. The profile was characterized by multiple, large-scale segmental duplications and deletions:

**TABLE 1:** Patient hematology test results.

Test	Result	Unit	Reference range
Total protein (TP)	56	g/L	65–85
Albumin (Alb)	33	g/L	40–55
Globulin (Glo)	23	g/L	20–24
Albumin/globulin ratio	1.43	mg/L	1.2–2.4
Prealbumin (PA)	100	mg/L	180–350
Aspartate aminotransferase (AST)	303	IU/L	13–35
Alanine aminotransferase (ALT)	84	IU/L	7–40
Alkaline phosphatase (ALP)	491	IU/L	35–100
Gamma-glutamyl transferase (GGT)	414	IU/L	7–45
Total bilirubin (T-Bil)	32	μmol/L	0–21
Direct bilirubin (D-Bil)	27	μmol/L	0–8
Indirect bilirubin (I-Bil)	5	μmol/L	0–17
Total bile acids (TBA)	31	μmol/L	0.5–10
Alpha-fetoprotein (AFP)	>20,000	ng/mL	0–13.4
Carbohydrate antigen CA-153	30.5	U/mL	0–31.3
Human epididymis protein 4 (HE4)	26.9	pmol/L	20–1500

Duplications: 1q21.1-q44(∼102.9 Mb), 4p16.3-q11(∼49.8 Mb), 6p25.3-p11.2(∼60 Mb), 6q12-q16.2(∼23.1 Mb),8p12-p11.1(∼12.5 Mb),8q11.21-q21.12(∼12.5 Mb),10p15.3-p11.21(∼33.7 Mb), 10q11.21-q26.3 (∼89.4 Mb), 18q12.1-q12.3 (∼11.3 Mb), 20p13-p11.21 (∼29.8 Mb), and 20q11.21-q13.33 (∼38.5 Mb).

Deletions: 1p36.33-p31.3 (∼70 Mb), 4q13.1-q35.2 (∼87.6 Mb), 6q21-q27 (∼68.4 Mb), 8p23.3-p21.1(∼29 Mb). The key clinical events from the initial NIPT test to the final diagnosis in this case are summarized in a timeline in Table 2. This study was approved by the hospital ethics committee (2025Lunhan Examination No. 014).

**TABLE 2:** Clinical timeline from initial Non-Invasive Prenatal Testing (NIPT) to final diagnosis.

Gestational week	Clinical event
22 weeks	First NIPT performed Result: abnormal genome-wide CNV pattern detected
22 weeks	Repeat NIPT performed Result: confirmed abnormal CNV pattern
23 weeks	Amniocentesis performed Fetal karyotype: 46,XX (normal) Fetal CMA: no abnormalities detected
28–33 weeks	Asymptomatic period Regular prenatal follow-up
34 weeks + 3 days	Admitted due to upper abdominal pain, loss of appetite Physical examination: hepatomegaly, tenderness Laboratory findings: Markedly elevated alpha-fetoprotein (>20,000 ng/mL), abnormal liver function tests Ultrasound: multiple solid liver lesions Magnetic resonance imaging: findings consistent with multifocal hepatocellular carcinoma
34 weeks + 5 days	Clinical diagnosis: multifocal hepatocellular carcinoma (based on imaging and laboratory findings; pathological confirmation not obtained due to pregnancy)

## Discussion

Non-Invasive Prenatal Testing entails deep sequencing of cell-free fetal DNA (cffDNA) derived from maternal plasma, followed by bioinformatic analysis to ascertain fetal genetic status. cffDNA is predominantly released into the maternal circulation via apoptosis or necrosis of placental trophoblast cells and coexists with maternal-origin cell-free DNA (cfDNA). In uncomplicated pregnancies, cffDNA constitutes 5%–15% of total maternal cfDNA, with the remaining 85%–95% originating from maternal sources, including hematopoietic cells, vascular endothelial cells, and solid organs such as the liver (1, 2). In the presence of maternal malignancy, apoptotic or necrotic tumor cells release circulating tumor DNA (ctDNA) into the bloodstream. Chromosomal instability (CIN), a hallmark of malignancy, predisposes tumor cells to recurrent chromosomal numerical and structural aberrations, which are recapitulated in shed ctDNA (3). Consequently, NIPT, while primarily intended for fetal aneuploidy detection, may incidentally capture tumor-associated copy number variations (CNVs), enabling the potential identification of occult maternal malignancies (4). A growing body of literature has documented occult maternal malignancies incidentally detected through abnormal noninvasive prenatal testing (NIPT) results (Table 3). In 2013, Osborne et al. first reported a pregnant woman whose NIPT indicated trisomies 18 and 13 and who was postnatally diagnosed with metastatic small cell carcinoma (5). Bianchi et al. analyzed NIPT data from 125,426 pregnant women, identifying 3,757 cases with multiple chromosomal aneuploidies (MCA), 10 of whom were diagnosed with malignancy during pregnancy (6). In a more recent study, Turriff et al. found that among rigorously screened subjects with NIPT showing copy number variations (CNVs) involving multiple chromosomes, the cancer detection rate was 95.9%, spanning a range of cancer types including colorectal, breast, lung, and pancreatic cancers (7).

**TABLE 3:** A retrospective analysis of Non-Invasive Prenatal Testing (NIPT)-hinted malignancy during pregnancy.

References	Tumor type	NIPT copy number variation pattern	Time to diagnosis
Osborne et al. (5)	Metastatic small cell carcinoma (vaginal origin)	Trisomy 13 and trisomy 18 (multiple chromosomal aneuploidies)	Postpartum
Bianchi et al. (13)	Lymphoma (*n* = 7), leukemia (*n* = 1), breast cancer *(n* = 1), colorectal cancer (*n* = 1), leiomyosarcoma (*n* = 1), multiple myeloma (*n* = 1)	Genome-wide nonspecific CNV gains and losses, “sawtooth” pattern; multiple chromosomal aneuploidies (chromosomes 13, 18, 21, X, Y)	During pregnancy or postpartum
Anami et al. (14)	Metastatic melanoma	Trisomy 13; no reportable results for chromosomes 21 and 18	During pregnancy
Moellgaard et al. (15)	Unspecified malignancy	1q21.1-q44 gain, 3q11.2-q29 gain, chromosome 5 monosomy, 8p23.3-p21.1 loss, 8q11.21-q24.3 gain, 12p13.33-p11.1 gain, 12q12-q24.33 loss, 13q12.11-q34 gain, 14q11.2-q32.33 monosomy, 19p13.3-p13.11 gain, 19q13.11-q13.43 gain	During pregnancy
Heesterbeek et al. (11)	Primary mediastinal B-cell lymphoma, classical Hodgkin lymphoma, others	Genome-wide CNV abnormalities with multiple chromosomal gains and losses	During pregnancy or postpartum
Ibirogba et al. (16)	Intrahepatic cholangiocarcinoma (Stage IIIB)	“Atypical findings” (specific CNV not reported in detail)	Diagnosed at 25–28 weeks after symptom onset
Ibirogba et al. (16)	Intrahepatic cholangiocarcinoma (Stage IIIB); adrenocortical carcinoma (Stage III high-grade)	Atypical findings (specific CNV not reported in detail)	Diagnosed at 25–28 weeks (cholangiocarcinoma); adrenal mass detected by MRI at 25 weeks (adrenocortical carcinoma)
Turriff et al. (7)	Colorectal cancer, breast cancer, lung cancer, pancreatic cancer, others	Multiple chromosomal CNVs (≥3 chromosomal subchromosomal and/or whole chromosomal gains and losses)	During pregnancy or postpartum

In this study, the CNV profile identified in NIPT sequencing data bears striking resemblance to the chromosomal instability signature characteristic of hepatocellular carcinoma (HCC). The 1q21.1-q44 duplication (∼102.9 Mb) mirrors the most prevalent chromosomal gain in HCC, reported in 58%–86% of tumors and harboring oncogenes such as CHD1L and ARNT that drive cell cycle progression. Similarly, the 8q amplification corresponds to the second most frequent gain in HCC (∼50% incidence), encompassing the MYC locus and genes involved in metastatic dissemination (8). Notably, the 8p23.3-p21.1 deletion (∼29 Mb) parallels one of the most clinically significant losses in HCC, occurring in 52%–65% of patients and associated with decreased survival through haploinsufficiency of tumor suppressor genes including DLC1 (9). Recent plasma cfDNA-based studies have successfully captured these exact alteration patterns in HCC patients, with arm-level amplifications of 1q and 8q and deletions of 8p emerging as recurrent signatures across diverse cohorts (8). The concordance between our NIPT CNV profile and the established HCC genomic landscape suggests that the genome-wide chromosomal instability detected at 22 weeks gestation likely originated from the patient’s occult multifocal HCC, which became clinically apparent 12 weeks later. This temporal sequence—where NIPT aberrations preceded radiological and biochemical diagnosis—demonstrates the potential of cfDNA analysis to serve as an early sentinel event for maternal malignancy during pregnancy. Furthermore, the presence of chronic hepatitis since childhood in this patient represents a known risk factor for HCC development, and the specific CNV pattern we identified is consistent with the complex karyotypic abnormalities typically observed in HBV-associated hepatocarcinogenesis (8). These findings support the integration of NIPT-based cfDNA CNV profiling into expanded maternal screening protocols, particularly for high-risk populations with chronic liver disease, enabling earlier detection and intervention for occult malignancies. A key limitation of this study is the lack of direct genomic confirmation from tumor tissue. Although the strong concordance between the complex CNV pattern in maternal cfDNA and the subsequent diagnosis of multifocal HCC strongly suggests a causal link, definitive evidence would require comparative CNV analysis between tumor tissue and cfDNA to rule out other sources such as placental mosaicism, benign tumors, or clonal hematopoiesis. However, due to the patient’s pregnancy and the multifocal nature of HCC, tumor biopsy prior to delivery was not feasible. Thus, while highly suggestive, the association remains inferential based on clinical, imaging, and laboratory correlations. Future studies should aim to obtain tissue confirmation whenever clinically possible to establish definitive causality.

This case highlights a critical challenge in prenatal diagnosis: how to interpret abnormal NIPT results when fetal evaluation is normal. Current approaches to the clinical management of malignancy during pregnancy predominantly support the establishment of a multidisciplinary clinical management pathway, involving genetic counselors, obstetricians, oncologists, radiologists, pathologists, and neonatologists to collectively perform risk assessment and interpret genetic laboratory findings (10).

### Initial identification and confirmation

When NIPT reveals multiple copy number variations (CNVs) or multiple chromosomal aneuploidies (MCA), repeat blood sampling for confirmation is the first recommended step. If the repeat results are consistent with the initial findings, a high suspicion for maternal malignancy should be raised, with involvement of multiple chromosomal aberrations carrying greater clinical significance than isolated variants (11). Additionally, other potential causes of chromosomal aberrations should be excluded, including benign tumors (e.g., uterine leiomyomas), vitamin B12 or folate deficiency, and autoimmune disorders such as systemic lupus erythematosus (12).

#### Fetal evaluation

Because tumor-derived circulating tumor DNA (ctDNA) may mask fetal chromosomal signals and compromise the accurate assessment of fetal chromosomal abnormality risk, further ultrasonographic evaluation should be performed to assess for fetal growth restriction or congenital structural anomalies. When indicated, invasive prenatal diagnosis via amniocentesis or chorionic villus sampling should be pursued to definitively determine the fetal chromosomal status.

### Maternal evaluation

If both initial and repeat NIPT results demonstrate multiple CNVs or MCA, genetic counselors and obstetricians should promptly convene a multidisciplinary team including oncologists, radiologists, and pathologists to develop an individualized plan for subsequent evaluation and intervention. Maternal evaluation should include:

History and physical examination: Comprehensive history taking, with physical examination focused on the breasts, uterus, thyroid, lymph nodes, liver, spleen, and skin.

#### Gynecologic evaluation

Routine gynecologic examination and ultrasound to exclude uterine leiomyomas or gynecologic malignancies.

#### Laboratory studies

Complete blood count, liver and renal function tests, glucose and lipid panel, electrolytes and acid-base balance, and tumor marker testing.

#### Imaging

Magnetic resonance imaging (MRI), computed tomography (CT), or other modalities as clinically indicated.

### Postpartum follow-up

If the specific etiology of the abnormal NIPT results remains unidentified during pregnancy, repeat NIPT is recommended within 1–6 weeks postpartum. If repeat testing yields normal results, no further evaluation for maternal malignancy is required. If chromosomal aberrations persist, the patient should be referred to oncology for reassessment, with repeat imaging and laboratory studies (10). If no malignancy is identified after such evaluation, annual complete blood count monitoring may be considered.

## Conclusion

From a technical perspective, the development of predictive models using NIPT data can incorporate multi-dimensional biological features, including but not limited to ctDNA copy number variations, fragment size profiles, and methylation patterns. Such integrated approaches, combined with standardized clinical protocols and multidisciplinary collaboration, hold promise for improving early detection of occult maternal malignancies during pregnancy.

Future research directions should focus on: establishing large prospective cohorts to determine the positive predictive value of various NIPT abnormality patterns for maternal malignancies; developing standardized reporting guidelines for NIPT laboratories to enable consistent identification and communication of findings suspicious for maternal cancer; validating tumor-derived CNV signatures that may predict specific cancer types, enabling more targeted diagnostic workups; and integrating NIPT-based ctDNA analysis with other maternal biomarkers to develop robust predictive models that balance sensitivity, specificity, and clinical utility. Ultimately, these efforts will facilitate the translation of incidental NIPT findings into actionable clinical pathways that optimize maternal and fetal outcomes.

## Data Availability

The datasets presented in this study can be found in online repositories. The names of the repository/repositories and accession number(s) can be found in the article/supplementary material.

## References

[1] Nygren AO Dean J Jensen TJ Kruse S Kwong W van den Boom D et al. Quantification of fetal DNA by use of methylation-based DNA discrimination. *Clin Chem.* (2010) 56:1627–35. 10.1373/clinchem.2010.146290 20729299

[2] Hui L Bianchi DW. Fetal fraction and noninvasive prenatal testing: what clinicians need to know. *Prenat Diagn.* (2020) 40:155–63. 10.1002/pd.5620 31821597 PMC10040212

[3] Golara A Kozłowski M Cymbaluk-Płoska A. The role of circulating tumor DNA in Ovarian Cancer. *Cancers.* (2024) 16:3117. 10.3390/cancers16183117 39335089 PMC11430586

[4] Arisi MF Dotan E Fernandez SV. Circulating tumor DNA in precision oncology and its applications in Colorectal Cancer. *Int J Mol Sci.* (2022) 23:4441. 10.3390/ijms23084441 35457259 PMC9024503

[5] Osborne CM Hardisty E Devers P Kaiser-Rogers K Hayden MA Goodnight W et al. Discordant noninvasive prenatal testing results in a patient subsequently diagnosed with metastatic disease. *Prenat Diagn.* (2013) 33:609–11. 10.1002/pd.4100 23559449

[6] Lenaerts L Jatsenko T Amant F Robert Vermeesch J. Noninvasive prenatal testing and detection of occult maternal malignancies. *Clin Chem.* (2019) 65:1484–6. 10.1373/clinchem.2019.306548 31604758

[7] Turriff AE Annunziata CM Malayeri AA Redd B Pavelova M Goldlust IS et al. Prenatal cfDNA sequencing and incidental detection of maternal Cancer. *N Engl J Med.* (2024) 391:2123–32. 10.1056/NEJMoa2401029 39774314 PMC11711700

[8] Wang Y Zhou K Wang X Liu Y Guo D Bian Z et al. Multiple-level copy number variations in cell-free DNA for prognostic prediction of HCC with radical treatments. *Cancer Sci.* (2021) 112:4772–84. 10.1111/cas.15128 34490703 PMC8586684

[9] Shahrisa A Tahmasebi-Birgani M Ansari H Mohammadi Z Carloni V Mohammadi Asl J. The pattern of gene copy number alteration (CNAs) in hepatocellular carcinoma: an in silico analysis. *Mol Cytogenet.* (2021) 14:33. 10.1186/s13039-021-00553-2 34215297 PMC8254242

[10] Heesterbeek CJ Lenaerts L Tjan-Heijnen VCG Amant F van Rij MC Theunis M et al. Comprehensive recommendations for the clinical management of pregnant women with noninvasive prenatal test results suspicious of a maternal malignancy. *JCO Oncol Pract.* (2024) 20:1027–34. 10.1200/OP.23.00594 38608208

[11] Heesterbeek CJ Aukema SM Galjaard RH Boon EMJ Srebniak MI Bouman K et al. Noninvasive prenatal test results indicative of maternal malignancies: a nationwide genetic and clinical follow-up study. *J Clin Oncol.* (2022) 40:2426–35. 10.1200/JCO.21.02260 35394817

[12] Schuring-Blom H Lichtenbelt K van Galen K Elferink M Weiss M Vermeesch JR et al. Maternal vitamin B12 deficiency and abnormal cell-free DNA results in pregnancy. *Prenat Diagn.* (2016) 36:790–3. 10.1002/pd.4863 27328203

[13] Bianchi DW Chudova D Sehnert AJ Bhatt S Murray K Prosen TL et al. Noninvasive prenatal testing and incidental detection of occult maternal malignancies. *JAMA.* (2015) 314:162–9. 10.1001/jama.2015.7120 26168314

[14] Anami Y Minami S Kumegawa A Matsukawa H Nishioka K Noguchi T et al. Malignant melanoma treated with pembrolizumab during pregnancy: a case report and review of the literature. *Mol Clin Oncol.* (2021) 15:242. 10.3892/mco.2021.2404 34650809 PMC8506525

[15] Moellgaard MH Lund ICB Becher N Skytte AB Andreasen L Srebniak MI et al. Incidental finding of maternal malignancy in an unusual non-invasive prenatal test and a review of similar cases. *Clin Case Rep.* (2022) 10:e6280. 10.1002/ccr3.6280 36245448 PMC9552546

[16] Ibirogba ER Macdonald E Tsimis ME Cordoba M Thakur M Romero VC. Incidental diagnosis of occult maternal malignancy with routine noninvasive prenatal testing during pregnancy. *Cureus.* (2024) 16:e76331. 10.7759/cureus.76331 39850155 PMC11756998

